# Development of A Loop-Mediated Isothermal Amplification (LAMP) Assay for Detection of Relapsing Fever Borreliae

**DOI:** 10.18502/jad.v14i1.2703

**Published:** 2020-03-31

**Authors:** Faezeh Houmansadr, Mohammad Soleimani, Saied Reza Naddaf

**Affiliations:** 1 Department of Cellular and Molecular Biology, Science and Research Branch, Islamic Azad University, Tehran, Iran; 2 Department of Microbiology, Faculty of Medicine, AJA University of Medical Sciences, Tehran, Iran; 3 Tasnim Biotechnology Research Center, Faculty of Medicine, AJA University of Medical Sciences, Tehran, Iran; 4 Department of Parasitology, Pasteur Institute of Iran, Tehran, Iran

**Keywords:** Relapsing fever, LAMP, *glpQ*, Iran

## Abstract

**Background:**

This study aimed to develop a loop-mediated isothermal amplification (LAMP) assay for the rapid detection of tick-borne relapsing fever in resource-limited areas.

**Methods:**

A set of six primers were designed based on the conserved regions of the Glycerophosphodiester phosphodiesterase (*glpQ*) gene of *Borrelia* species. For sensitivity assay, serial dilutions of a recombinant plasmid containing a 219bp sequence of the glpQ were prepared and used as the template DNA. The LAMP reactions containing the six primers and the reagents required for amplification were incubated at 60–65 °C for 60min in a Loopamp real-time turbidimeter. For the specificity test, DNA from 14 other bacteria were included in the assays, and double-distilled water was used as the negative control. Also, DNA from dried blood spots (DBSs) of spirochetemic mice, and blood samples from relapsing fever-suspected patients were examined by the LAMP along a *Borrelia*-specific nested PCR that targets the *rrs-rrl-*IGS region.

**Results:**

The LAMP detected as low as 90*glpQ* copies in reactions. The primers reacted with DNA from DBS of spirochetemic mice showing spirochete concentrations of ≤ one per a 1000X microscopic field. In clinical samples, the LAMP assay showed a higher sensitivity compared to nested-PCR. The LAMP specificity was 100%, as the primers did not react with other bacteria DNA.

**Conclusion:**

The high sensitivity and specificity of the test, along with the simplicity of the DNA extraction procedure, make the LAMP a reliable and adaptable tool for the diagnosis of tick-borne relapsing fever in rural endemic areas.

## Introduction

The genus *Borrelia* comprises two distinct groups of spirochetes with the difference in diseases they cause. One group includes the causative agents of Lyme disease and the other the relapsing fever borreliae (RFB). Currently, there are 22 confirmed RFB, and six other taxa have been proposed (1). Except for the louse adapted *Borrelia recurrentis*, the majority of species pathogenic to humans are transmitted by the soft ticks of the genus *Ornithodoros*. A few species, such as *Borrelia miyamotoi* and *Borrelia lonestari*, are vectored by hard ticks and yet share genetic similarities with the RFB (2). Soft tick-borne relapsing fever (STBR) is endemic to Iran (3–5). Until now, despite the improvement of housing and the removal of the disease from mandatory reporting to the Ministry of Health and Medical Education (MHME), no year has passed without reports of human infections. In Iran, four RFB, including *Borrelia persica*, *Borrelia microti*, *Borrelia latyschewii* and *Borrelia baltazardi* have been described (5). *Borrelia persica* is the primary cause of the disease especially in the west and northwest of the country (5–7), while in the south, epidemiological data and molecular approved human infections indicated *B. microti* and other *B. microti*-like borreliae as the other cause of relapsing fever (3, 8, 9).

In Iran, until recently, confirmation of relapsing fever merely relied on observation of the spirochetes in peripheral blood of febrile patients using darkfield microscopy or Giemsa-stained blood smears. The disease is easily diagnosed by microscopy during fever peaks with a massive spirochetemia. However, between the peaks and in milder diseases, the bacteria are scanty and are hard to identify in blood smears. In Iran, PCR assays using various molecular markers like *flaB*, *glpQ* and *rrs* have successfully detected the *Borrelia* spp in relapsing fever patients (3, 8) and animals (10). The PCR assays exhibit high sensitivities but are not commonly affordable in resource-limited laboratories of rural areas, where most of the relapsing fever infections occur. Hence, we prompted to develop an alternative DNA amplification assay of lower cost for the detection of the disease in these areas. Loop-mediated isothermal amplification (LAMP) has proved as a robust, cheap, highly sensitive/specific tool for the detection of various pathogenic agents including viruses, bacteria, fungi, and parasites (11–14). This assay employs a DNA polymerase and a set of 4–6 primers that operate in isothermal conditions forming loop structures that ultimately precipitate in the reaction mixture (13). This assay has also shown to be less prone to inhibition from DNA preparations and allows adaptability to field conditions (15, 16).

This study aimed to develop a (LAMP) assay for the rapid detection of relapsing fever borreliae based on the glycerophosphodiester phosphodiesterase (*glpQ*) gene, a sequence conserved among all relapsing fever borreliae, but absent from Lyme disease spirochetes (17).

## Materials and Methods

### Bacteria species and DNA extraction

We used *B. microti* strain IR-1, which was maintained via continual passages in NMRI mice for more than 15 years in the Parasitology Department of Pasteur Institute of Iran. Blood samples were obtained from *B. microti*-infected mice when spirochetes reached 1.4× 10^6^/ml of blood. DNA extraction from 1ml of blood was performed using a Genomic DNA Purification Kit (Promega, Madison, USA) as described by the manufacturer.

### Primer design

**Fig. 1.**
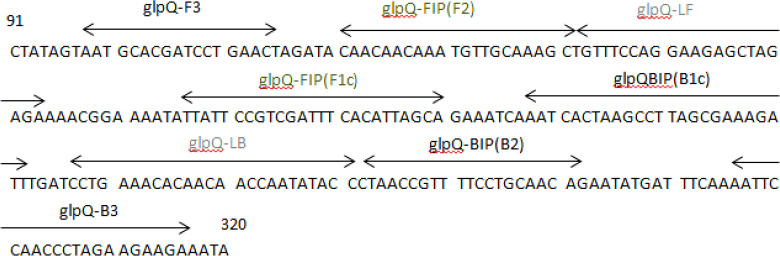
The regions of the *glpQ* from which the primers designed for LAMP assay. Forward outer primer, glpQ-F3; backward outer primer, glpQ-B3; forward inner primer, glpQ-FIP (F2)+glpQ-FIP (FIc); backward inner primer, glpQ-BIP (B2)+glpQ-BIP (BIc); forward loop primer, glpQ-LF; backward loop primer, glpQ-LB

Initially, glpQ sequences of 10 relapsing fever *Borreliae* (*B. microti*, Acc. No. JF825473; *B. microti*, Acc. No. EU914144; *B. recurrentis*, Acc. No. KJ003842, *Borrelia* sp., Acc. No. KX683865; *Borrelia duttonii*, Acc. No. DQ 346785; *Borrelia crocidurae*, Acc. No. CP 004267; *Borrelia hispanica*; Acc. No. GU 357573; *B. persica*, Acc. No. EU914143; *B. recurrentis*, Acc. No. AF247152; *Borrelia duttonii*, Acc. No. GU357577) were obtained from the GenBank database and aligned by using CLC Sequence Viewer 7 (CLC bio, Aarhus, Denmark). A set of six primers including two loop primers were designed based on the conserved regions of the glpQ sequence, corresponding to the nucleotides 261015–261682 of *B. duttonii* strain Ly (Fig. 1), by the online software program, Primer Explorer V4 (Eiken Chemical Co., Tokyo, Japan; http://primerexplorer.jp/e/). The theoretical specificity of the designed primers was confirmed by in silico analysis using BLAST and Primer-BLAST software available in NCBI ( http://www.ncbi.nlm.nih.gov/). The primers were synthesized by a commercial company (Generay Biotechnology, Shanghai, China). The main primers (glpQ-F3 and glpQ-B3) and (glpQ-FIP and glpQ-BIP) target fragments of 219bp and 161bp size, respectively, and the loop primer (glpQ-LF and glpQ-LB) produce amplicons of various size with a ladder-like pattern (Table 1).

**Table 1.** The primers designed and used for amplification of *glpQ* gene by LAMP assay

**Primers**	**Name**	**Sequences (5′ to 3′)**
**glpQ-F3**	Forward outer primer	AATGCACGATCCTGAACT
**glpQ-B3**	Backward outer primer	TCTTCTTCTAGGGTTGGAATT
**glpQ-FIP**	Forward inner primer	TGCTAATGTGAAATCGACGGAATAA-CAACAACAAATGTTGCAAAGC
**glpQ-BIP**	Backward inner primer	AATCACTAAGCCTTAGCGAAAGAT-TGTTGCAGGAAAACGGTTA
**glpQ-LF**	Forward loop primer	TCTCTAGCTCTTCCTGGAAACA
**glpQ-LB**	Backward loop primer	CCTGAAACACAACAACCAATATACC

### Glycerophosphodiester Phosphodiesterase Gene (*glpQ*) cloning

A 219bp fragment of the glpQ gene was amplified by the primers *glpQ*-F3 and glpQ-B3 (Table 1). The reaction contained 3mM MgSO_4_ (Biobasic, Toronto, Canada), 1.6mM dNTPs (Kawsar Biotech Co, Tehran, Iran), 1μl 10X buffer [100mM KCl, 100mM (NH4)2SO4, 200mM Tris HCl (pH 8.75)], 1% Triton X-100, 1mg/ml BSA (Biobasic, Toronto, Canada), 1U Taq DNA Polymerase (Biobasic, Toronto, Canada), 0.4μM of each primer, 1μl template DNA, and double-distilled water (DDW) to the 25μl final volume. The amplification was programmed in a thermal cycler (Eppendorf, Hamburg, Germany), for an initial denaturation at 94 °C for 4min followed by 35 cycles of 94 °C for 45sec, 45 °C for 45sec, and 72 °C for 30sec, and a final extension at 72 °C for 10min. The PCR products were run on a 2% agarose gel (Min Run Gel Electrophoresis System; Bio-Equip co, Shanghai, China), stained with ethidium bromide (CinnaGen, Alborz, Iran), and visualized under UV in Gel Documentation system (E-BOX VILBER, Marne*-*la*-*Vallée, France). The PCR product was purified using a PCR Purification Kit (Bioneer, Daejeon, South Korea), cloned into a TA vector (InsTAclone™ PCR Cloning Kit, Thermo Scientific, MA, United States), and transformed in *Escherichia coli* Top10F’. The bacteria were incubated at 37 °C for 24h on Luria-Bertani medium (Merck, KGaA, Darmstadt, Germany) containing 24mg/ml IPTG (isopropyl-beta-D-thiogalactopyranoside) (Fermentas, Ontario, Canada), 20mg/ml X-gal (5-bromo-4-chloro-3-indolyl beta d-galactoside) (Fermentas, Ontario, Canada), 10mg/ml tetracycline (Razak, Alborz, Iran) and 50mg/ml ampicillin (Cosar, Tehran, Iran). The recombinant bacteria were identified by blue/white screening, with the while colonies representing recombinant ones. One white colony was added to Luria-Bertani broth medium containing 10mg/ml tetracycline and 50mg/ml ampicillin followed by incubation at 37 °C for 16h while shaking at 180 RPM. Plasmid purification was performed by the AccuPrep Plasmid Mini Extraction kit (Bioneer, Daejeon, South Korea) and the presence of the *glpQ* gene in the recombinant plasmids was confirmed by PCR amplification with the primers glpQ-F3 and glpQ-B3. The recombinant plasmid was named pTZ57R/T-glpQ.

### Sensitivity assay

A serial 10-fold dilution of the recombinant plasmid ranging from 9×10^8^ to 9×10^−1^ copy numbers per microliter, equivalent to ≈32ng to ≈32×10^4^ fg/μl of DNA was prepared and used in assays.

### Specificity assay

For specificity assays, we used DNA of 14 other bacteria including *Shigella sonnei* ATCC 9290, *Klebsiella pneumoniae* ATCC 7881, *Bacillus subtilis* ATCC 6051, *Staphylococcus aureus* ATCC 25923, *Enterococcus faecalis* ATCC 29212, *Enteropathogenic Escherichia coli* (EPEC) ATCC 43887, *Yersinia enterocolitica* ATCC 23715, *Pseudomonas aeruginosa* ATCC 27853, clinical specimens of *Escherichia coli*, *Salmonella typhi*, *Acinetobacter baumannii*, *Citrobacter* sp, *Enterobacter* sp, and *Leptospira interrogans* in all assays. All the bacteria, including *L. interrogans*, the closely related bacteria to *Borrelia* species contained glpQ sequence.

### Loop Mediated Isothermal Amplification (LAMP) assay

The LAMP reactions contained 40pM of the inner (glpQ-FIP and glpQ-BIP) and 10pM of outer (glpQ-F3 and glpQ-B3) and loop primers (glpQ-LF and glpQ-LB) (Table 1), 11.2 mM dNTPs (Kawsar Biotech Co, Tehran, Iran), 0.8M betaine (Sigma Aldrich, Taufkirchen, Germany), 20mM Tris-HCl, 10mM KCl, 10mM (NH _2 _)SO _4_ , 0.05% Triton X-100 (pH 8.8) (Biolabs, New England, UK), 8mM MgSo _4_ (Biobasic, Toronto, Canada), 0.1% Tween 20 (Acros Organics, Vernon, USA), 8U of Bst DNA polymerase, large fragment (Biolabs, New England, UK), 1μl of serial dilutions of the recombinant plasmid, and DDW to the 25 μl final volume. The reactions were incubated at temperatures ranging from 60–65 °C for 60min in a Loopamp real-time turbidimeter (LA-320C; Teramecs, Kyoto, Japan), followed by heating at 80 °C for 5min for enzyme inactivation. In all assays, DNAs from 14 other bacteria were included and DDW was used as a negative control.

During the optimization of the assay, some amplifications were performed without the loop primers. Also, in some reactions, we added 1μl of Fluorescent detection reagent containing Calcein (Eiken Chemical co., Tokyo, Japan), an indicator of DNA amplification.

### Detection of LAMP products

The amplification in LAMP reactions was examined by 1) naked eye observation of white turbidity resulting from the accumulation of magnesium pyrophosphate, a by-product of the reactions, 2) a Loopamp real-time turbidimeter that records the optical density of reactions every 6sec at 650nm (the reactions were considered positive when the turbidity reached ≥ 0.1 within 60min), 3) the color change from orange to green, as an indication of DNA amplification, and 4) gel electrophoresis of amplicons on 2% agarose gels.

A LAMP product resulting from the 9×10^8^ dilution was purified using PCR Purification Kit (Bioneer, Daejeon, Korea) and sequenced in both directions by (ABI 3730xl/Bioneer 3730xl, Daejeon, Republic of Korea).

### Preparation of DBSs from *Borrelia*-infected mice

Amounts of 200μl blood from *B. microti*-infected mice with various degrees of the spirochetemia (225±61.34, 71±36.71, 3.3±1.63, 1.1±1.44, and 0.8±0.78 spirochetes per microscopic field) were dotted on 30 DNA banking cards (DBC) (Kawsar Biotech Co, Tehran, Iran). Blood from non-infected mice was used as controls on DBCs. The blood spots were allowed to dry at room temperature, and the DBCs were kept in the same condition until used.

### Loop Mediated Isothermal Amplification (LAMP) and PCR with DBS

Circles of 2mm from DBSs were cut and washed three times with DNA extraction buffer (provided by the manufacturer), followed by DDW. The circles were allowed to dry at room temperature and then used in LAMP and PCR assays as described above except that instead of template DNA, DBS circles were included in the reactions.

The sensitivity and specificity of the LAMP assay were calculated in comparison with microscopy as the gold standard assay using MedCalc (2018 MedCalc Software bvba) software available online (https://www.medcalc.org/calc/diagnostic_test.php).

### Loop Mediated Isothermal Amplification (LAMP) and nested-PCR amplification of clinical specimens

DNA was extracted from 39 sera of febrile patients residing in the relapsing fever endemic areas in the south and west of Iran using a commercial DNA extraction kit as described above. The DNA samples were examined by the LAMP, and a *Borrelia*-specific nested PCR that amplifies the *rrs-rrl*-IGS region using the outer primers F, 5′-GTATG TTTAGTGAGGGGGGTG-3′ and R, 5′-GG ATCATAGCTCAGGTGGTTAG-3′ and inner nested primers F, 5′-AGGGGGGTGAAGTC GTAACAAG-3′ and R, 5′-GTCTGATAAACC TGAGGTCGGA-3′ (18). This PCR has exhibited high sensitivity in detecting relapsing fever borreliae (3, 8, 19, 20). In all assays, for specificity test, DNAs from other bacteria species were included and DDW was used as a negative control. The LAMP reactions were checked for DNA amplification as described above, and the PCR products were resolved on 2% agarose gels, visualized under UV and photographed.

## Results

### Glycerophosphodiester Phosphodiesterase Gene (*glpQ*) cloning

Amplification of the *glpQ* sequence from the recombinant plasmid (pTZ57R/T-glpQ) with the primers glpQ-F3 and glpQ-B3yielded the expected 219bp indicating the insertion of this sequence in the plasmid.

### Loop Mediated Isothermal Amplification (LAMP) assay with recombinant pTZ57R/T-glpQ

**Fig. 2.**
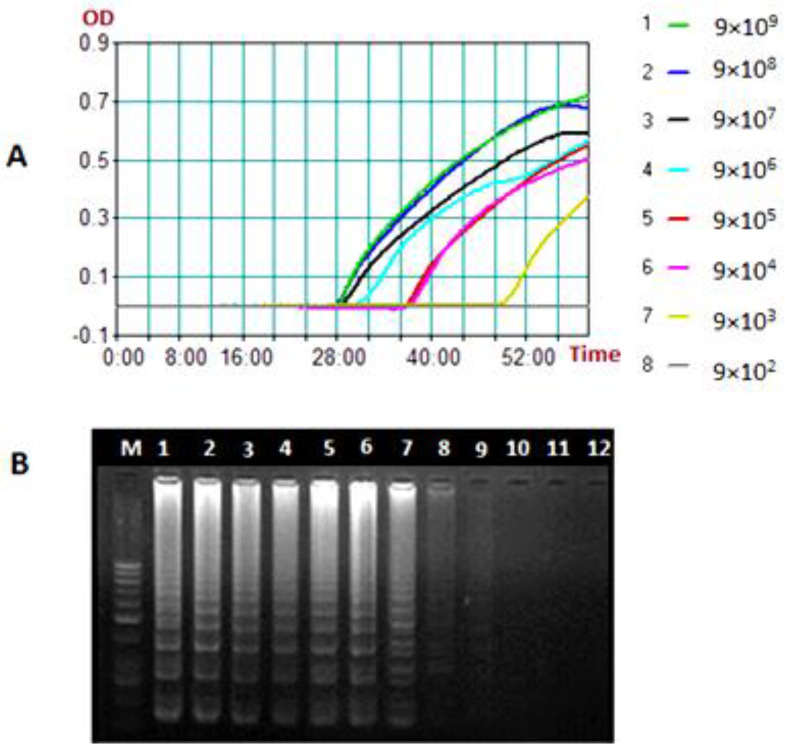
The sensitivity of the Loop Mediated Isothermal Amplification (LAMP) assay measured by a 10-fold serial dilution of a recombinant plasmid pTZ57R/T-glpQ plasmid ranging from 9×10^9^ to 9×10^−1^ /μl. A) The amplification curves generated by the Loopamp real-time turbidimeter, colored lines 1–7, serial dilutions 9×10^9^ , 9×10^8^ , 9×10^7^ , 9×10^6^ , 9×10^5^ , 9×10^4^ and 9×10^3^ ; line 8, serial dilutions ≤ 9×10^2^ . B) the LAMP products resolved on agarose gel, lane M, 50bp DNA ladder, lane 1, dilution 9×10^9^ ; lane 2, dilution 9×10^8^ ; lane 3, dilution 9×10^7^ ; lane 4, dilution 9×10^6^ ; lane 5, dilution 9×10^5^ ; lane 6, dilution 9×10^4^ ; lane 7, dilution 9×10^3^ ; lane 8, dilution 9×10^2^ ; lane 9, dilution 9×10^1^ ; lane 10, dilution 9×10^0^ ; lane 11, dilution 9×10^−1^ ; lane 12, negative control

We observed amplification of *glpQ* sequence in the LAMP reactions by naked eye observation of turbidity and the Loopamp real-time turbidimeter down to 9×10^3^ copies of recombinant plasmid equivalent to 0.32fg DNA (Fig. 2A). However, gel electrophoresis revealed amplifications in two lower dilutions of 9×10^2^ and 9×10^1^ , equivalent to 3.2fg and 0.32fg DNA, respectively (Fig. 2B). Our LAMP assay was 100% specific as the primers reacted with none of the other 14 bacterial DNA, including *L. introgans*. In the reactions containing calcein and the *Borrelia* DNA, the color turned from orange to green.

The 137bp sequence resulted following the sequencing of the LAMP amplicon matched with the *glpQ* sequence of *B. microti* strain IR-1 (acc. No. JF825473) corresponding to nucleotides 261158–261295 of the whole genome sequence of *B. duttonii* Ly (acc. No. CP000796). The results were the same within the temperature range of 60–65 °C. In the reactions, in abscence of loop primers, the optimum time for isothermal amplification was 60min, whereas, in those with the loop primers, the incubation time reduced to 45min.

### Loop Mediated Isothermal Amplification (LAMP) and PCR with DBSs

Of the 30 *B. microti*-positive DBS, all (100%) showed turbidity with the naked eye indicating amplification, while the glpQ-PCR (with the primers glpQ-F3 and glpQ-B3) yielded the expected 219bp band in 20 positive DBSs (66.67 %). The negative PCR reactions belonged to the mice with the lowest level of spirochetemia [1.1±1.44 (n= 4) and 0.8±0.78 (n= 6) spirochete per microscopic field]. Neither LAMP nor PCR amplification was observed with the negative controls (DDW) or reactions containing other bacteria DNA as the template.

By considering microscopy as the gold standard, the sensitivity of glpQ-LAMP and glpQ-PCR were 100% and 66.67%, respectively. The specificity was 100% for both assays.

### Loop Mediated Isothermal Amplification (LAMP) and PCR with clinical samples

**Fig. 3.**
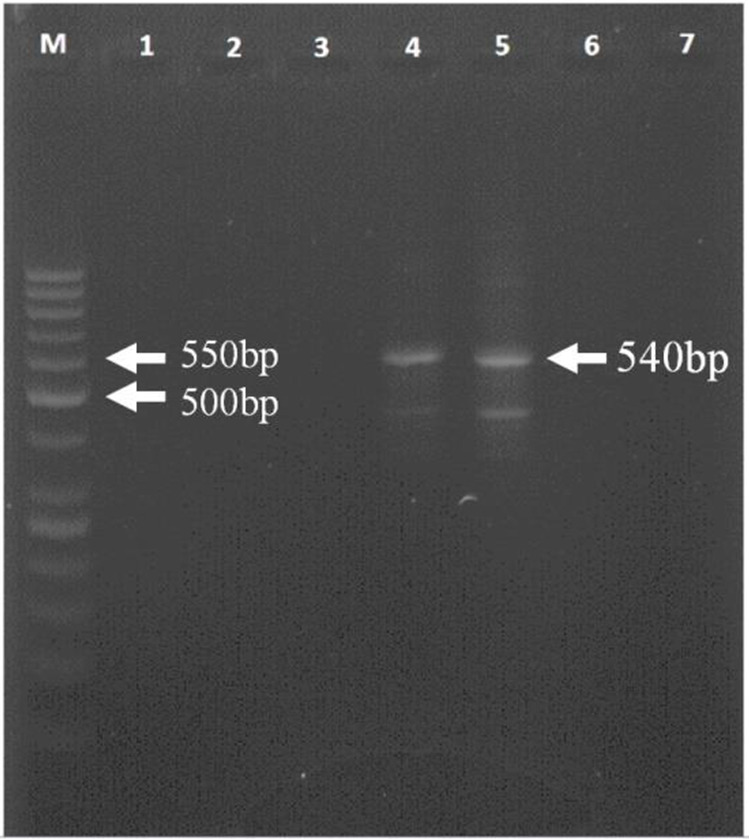
Gel electrophoresis of PCR amplification of the *rrs-rrl-*IGS region. Lane M, 5bp DNA ladder; lanes 1–6, human blood samples (a 540bp in lanes 4 and 5 indicates amplification of *Borrelia* DNA); lane 7, negative control (DDW)

Of 39 clinical samples, 11 became turbid in the LAMP assay indicating amplification of *glpQ* gene, whereas PCR amplification of IGS sequence only yielded the expected 540bp band in three specimens (Fig. 3).

## Discussion

In Iran, blood analysis of relapsing fever patients by examination of wet smears using dark-field microscopy or Giemsa-stained blood slides has been a common practice for years. This approach was efficient in endemic areas, where the disease commonly appeared in clusters (5), and overlooking the spirochetes in the blood of some febrile patients did not question the identity of the causative agent. However, with the decline of the disease in the endemic regions and reports of sporadic cases from other areas application of more sensitive approaches for the identification RFB became necessary. In Iran, over the past decade, PCR assays by based on various molecular markers, such as *glpQ*, *rrs*, and *flaB*, were developed for the identification of *Borrelia* infection in *Ornithodoros* ticks (21, 22) or characterization of the tick-originated relapsing fever borreliae (9, 23), but rarely clinical samples were included. Lately, qPCR and conventional PCRs identified a *B. microti*-like strain, presumably, an ecotype of African *B. duttonii*, in relapsing fever patients from southern Iran (3, 8).

In our previous work, using *B. persica*-spiked blood samples, we consistently observed bacteria by microscopy in the blood samples with densities ≥ 800–1000 spirochetes/μl (24). Herein, with the DBSs prepared with the spirochetemic murine blood, the LAMP could detect borrelial DNA in blood specimens showing less than one spirochetes per 1000X microscopic field. Our LAMP assay also showed a higher sensitivity in comparison with a nested-PCR amplification of the IGS in the diagnosis of clinical samples. The specificity of the LAMP assay for the diagnosis of *B. microti* DNA was 100% as the designed primers exhibited no cross-reaction with DNA from the other 14 bacteria used in this study. Our LAMP method requires to be further tested with blood samples from RF patients infected with other *Borrelia* species to ensure the sensitivity and specificity of the assay.

LAMP method has also shown promise for the detection of borreliae DNA and other pathogens in the tick vectors. In China, the LAMP could detect *B. burgdorferi* s. l. in ticks, with a higher sensitivity than a conventional PCR (25). Also, in recognizing spotted fever group rickettsia, the LAMP appeared ten times more sensitive than an end-point PCR targeting the same gene fragment (26).

## Conclusion

Such a reliable sensitivity and specificity, as well as the simplicity of the DNA extraction procedure, make the LAMP a suitable and adaptable assay for field studies in relapsing fever endemic areas. In resource-limited rural health centers, LAMP can readily check field-collected blood samples, preferably on DBS, for relapsing fever borreliae with the least equipment accessible, i.e., a heat block. Having access to a Loopamp real-time turbidimeter is preferable. However, LAMP is a qualitative assay, and the result can also be monitored by color change with calcein in the fluorescent detection reagent or by the turbidity of magnesium pyrophosphate in the reaction tubes. Hence, a heating system that provides a temperature within the range of 60–65 °C would suffice.
